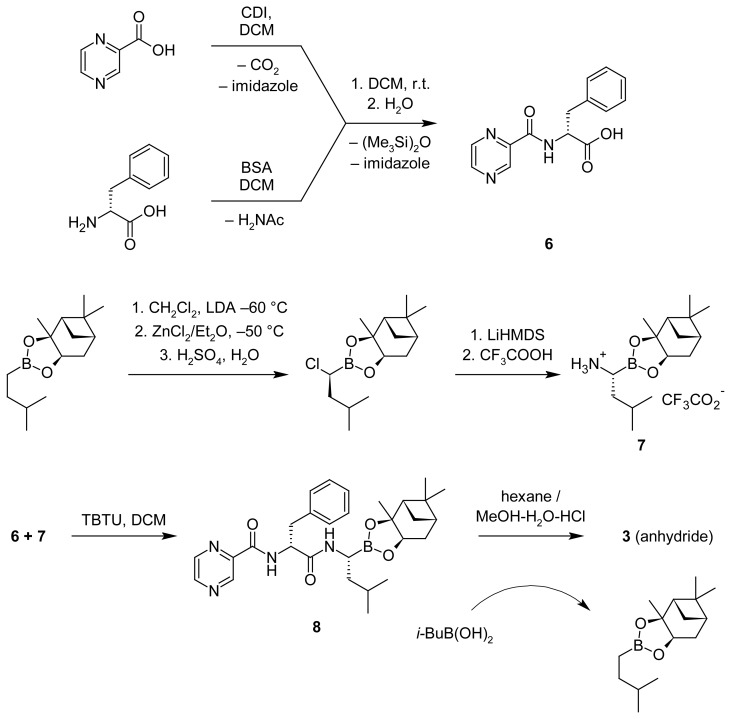# Synthesis and Characterization of Organic Impurities in Bortezomib Anhydride Produced by a Convergent Technology

**DOI:** 10.3797/scipharm.1109-02

**Published:** 2011-11-01

**Authors:** Andrey S. Ivanov, Sergey V. Shishkov, Anna A. Zhalnina

**Affiliations:** F-Synthesis, LLC, Posledniy Per., 11 (1), 107045 Moscow, Russia

**Keywords:** Bortezomib, Chemical synthesis, HPLC, Mass spectrometry, Impurities, Oxidation, Chirality, Velcade

## Abstract

A profile of impurities in bortezomib anhydride, produced by a recently developed convergent technology, has been characterized. HPLC-MS analysis of the drug essence revealed three impurities: an epimer of bortezomib, resulting from partial racemization of l-phenylalanine’s stereogenic center during the chemical synthesis, and two epimeric products of oxidative degradation of bortezomib, in which boron is replaced by the OH group. The impurities were obtained by chemical synthesis and characterized by physical methods.

## Introduction

**Fig. 1:**
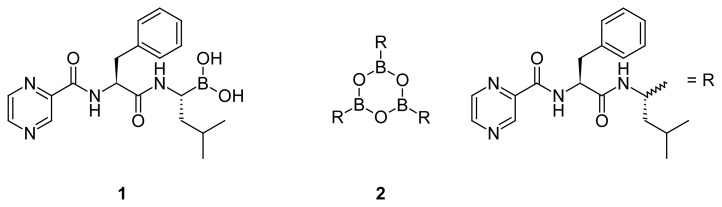


Bortezomib (Velcade) **1** (Fig. 1) is the first-in-class proteasome inhibitor anti-cancer drug. It is approved in the US for the treatment of relapsed multiple myeloma and mantle cell lymphoma [1]. Bortezomib acts as a highly selective reversible inhibitor of 26S proteasome, an ATP-dependent 2.4 MDa multicatalytic proteinaceous complex, regulating protein expression and function by degrading polypeptides and proteins, marked by ubiquitinilation. In neoplastic cells, proteosome inhibition may prevent degradation of pro-apoptotic factors, leading to cell death [2–5].

Bortezomib is available as a sterile lyophilized powder in single-dose vials, containing 3.5 mg of the active pharmaceutical ingredient and 35 mg of mannitol. The essence of bortezomib is produced in the form of trimeric boronic anhydride **2**. During the formulation, mannitol reacts with boroxine **2** to form the corresponding stable monomeric diester. The latter is hydrolyzed to release the active boronic acid **1** on reconstitution of the drug in saline solution for injection [6].

## Results and discussion

**Fig. 2:**
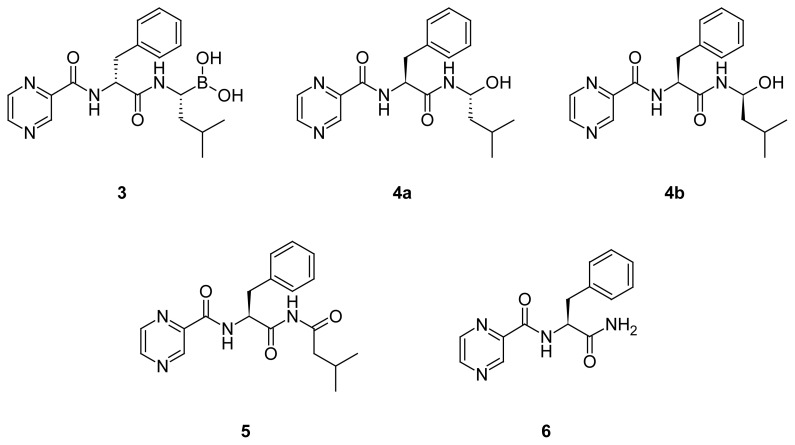


Earlier it was shown that oxidative degradation of commercially available bortezomib-mannitol adduct in a sterile 0.9% solution of NaCl in deuterated water led to formation of two products **4a** and **5** as identified by NMR, ESI-MS, and MALDI spectrometry [7]. In another study, treatment of boronic acid **1** with hydrogen peroxide in 1.1 mM aqueous solution at 20 °C gave rise to alcohol **4a**, retaining absolute configuration of the parent molecule. However, when oxidation was carried out in PEG300 : EtOH : H_2_O (4:1:5), initial oxidation product **4a** was undergoing a rapid isomerization to **4b** and further degradation to amide **6**. Also, acidic conditions (pH 1–2) accelerated isomerization of **4a**; in contrast, alkaline conditions (pH 11–12) led to quantitative transformation of **4a** into amide **6** [8] (Fig. 2).

**Fig. 3:**
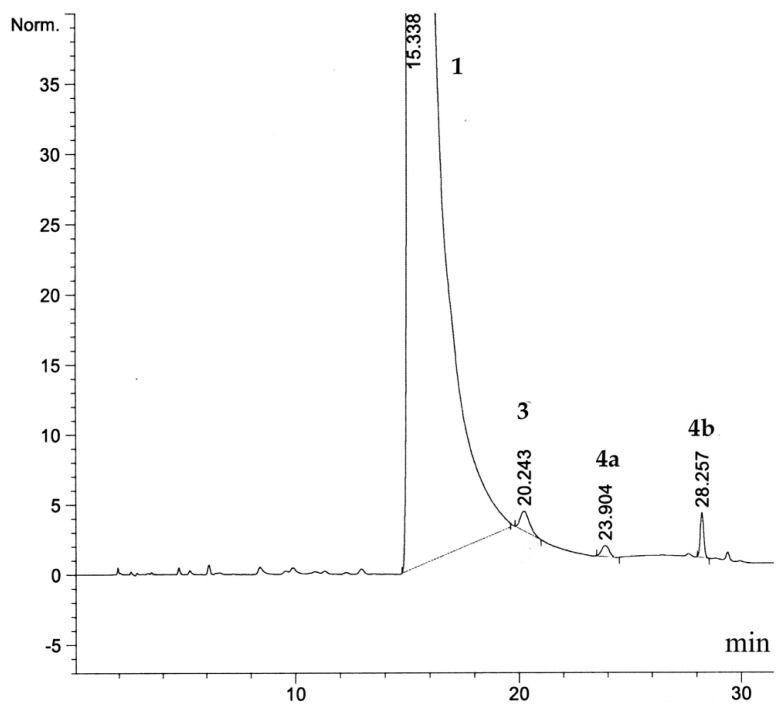


Recently, we reported a new efficient synthesis of bortezomib anhydride **2** employing a convergent approach [9]. According to the ICH guidelines (25 October 2006), all the impurities that exceed the identification threshold of 0.1% are to be characterized [10]. To comply with these recommendations, we investigated the profile of impurities in the bortezomib anhydride essence, produced by the abovementioned method. HPLC-MS analysis of 99.6% pure essence **2** revealed three organic impurities: **3**, **4a**, and **4b** in the ratio of 0.16, 0.13 and 0.07 % (Fig. 3).

Obviously, impurity **3** originates from partial racemization of l-phenylalanine’s chiral center during the chemical synthesis of bortezomib. It was shown that the use of TBTU in the fragment condensation between *N*-pyrazinecarbonyl-l-phenylalanine and an amino component resulted in a low degree of racemization as compared to *N*,*N*′-carbonyldiimidazole (CDI) [9]. However, even on activation with TBTU, the stereochemical integrity of the carboxy component is lost to some extent. This process is believed to proceed via an oxazolone intermediate (Scheme 1) and is favored by polar solvents and electron-withdrawing *N*^α^-acyl groups, such as pyrazinoyl [11].

Impurities **4a,b** are the products of degradation of boronic acid **1** or trimeric anhydride **2**, which arise because of oxidation of B–C bond with atmospheric oxygen. Oxidative deboronation is also the principal biotransformation pathway of bortezomib in humans [12].

To obtain compounds **3** and **4a,b** in the essentially pure form and in an amount sufficient for characterization, study of properties and use as references in chromatographic analysis, we performed their synthesis. Thus, epi-bortezomib **3** was synthesized starting from d-isomer of phenylalanine (Scheme 2). d-Phe was silylated with *N*,*O*-bis(trimethyl-silyl)acetamide (BSA) to give the corresponding *N*,*O*-bis-silyl derivative. The latter was reacted with pyrazinecarboxylic acid imidazolide, generated from pyrazinecarboxylic acid and CDI, to give *N*-pyrazinecarbonyl-d-phenylalanine **6** after aqueous work-up. (1*S*,2*S*,3*R*,5*S*)-pinanedioxy-*N*-pyrazinoyl-d-phenylalanyl-l-leucineboronate **8**, was synthesized according to a previously developed procedure [9]. Fragment condensation between carboxylic acid **6** and amine **7** was achieved using TBTU as a coupling agent. (1*S*,2*S*,3*R*,5*S*)-Pinanediol ester of epi-bortezomib **8** was isolated and transformed into epi-bortezomib **3** by an exchange reaction with isobutylboronic acid under strongly acidic conditions in a biphasic mixture of aqueous methanol-hexane. The (1*S*,2*S*,3*R*,5*S*)-pinanediol chiral auxiliary was recovered as the corresponding isobutylboronate. After crystallization from ethyl acetate compound **3** was obtained in the form of trimeric anhydride (similar to bortezomib anhydride **2**).

To obtain degradants **4a,b**, we carried out oxidation of boroxine **2** with aqueous hydrogen peroxide in methanolic solution. After evaporation of the solvent, both products were obtained in the essentially pure form as a 39:61 mixture of diastereomeres. LC-MS analysis showed similar main peaks for each diastereomer with m/z 339.4, corresponding to dehydrated molecular peaks [M − H_2_O + H]^+^. Therefore, the molecular weights were 356. Even molecular masses indicated that the molecules’ backbones, containing even number of nitrogen atoms, were intact. On the other hand, the absence of boron atom in the molecules was seen from the absence of characteristic boron isotope pattern (20% ^10^B and 80% ^11^B). Separation of **4a** and **4b**, prepared from monomeric boronic acid **1** using isocratic reverse phase HPLC, was reported by Wu, *et al*.; ^1^H and ^13^C NMR spectra of the diastereomeric mixture were in accordance with those reported for the separate diastereomers [8].

## Experimental

### Chemicals

Bortezomib anhydride (**2**) and (1*S*,2*S*,3*R*,5*S*)-pinanedioxy-(*R*)-1-ammonium-3-methylbutane-1-boronate trifluoracetate (**7**) were synthesized according to the previously developed procedures [9]. All the reagents and solvents were obtained from commercial sources. Diethyl ether was distilled from sodium benzophenone ketyl. Argon was dried by passing through a drying column.

### Equipment

Melting points were measured in open capillary tubes with OptiMelt MPA100 instrument. Optical rotations were determined on AA-55 Polarimeter at 20 °C. NMR spectra were recorded with an Avence II 600 and Bruker AMX-360 spectrometers. Chemical shifts were quoted in parts per million downfield from Si(CH_3_)_4_ (δ=0 ppm).

### HPLC and HPLC-MS analysis

HPLC analysis was performed at room temperature (*ca.* 20 °C) on Agilent 1200 instrument with UV detection at 270 nm. Chromatographic separation was carried out in a C_18_ 250×4.6 mm column (Knauer, Kromasil 100-5 sorbent). Flow rate was kept at 1.0 mL/min. Mobile phases were A (acetonitrile-water-HCOOH 300:700:1 v/v/v) and B (acetonitrile-water-HCOOH 800:200:1). Elution mode was 100% A during 5 min, gradient of B 0→100% during 15 min, and gradient of B 100→0 during 2 min. LC-MS analysis was performed on Waters Quattro API instrument with 2695 separation module and a diode array detector, working in the range of 220–500 nm. The HPLC system was coupled with a quadrupole mass spectrometer with an electrospray ionization (ESI) source, operating in the positive mode. Chromatographic separation was carried out in a 100×1 mm C_18_ XTerra column (Waters Co., 3.5 μm) using a linear gradient elution of eluents A (0.1%, v/v TFA in water) and B (0.1%, v/v TFA in acetonitrile). Flow rate was set at 1.0 mL/min.

### Synthesis

#### *N^α^*-(Pyrazin-2-ylcarbonyl)-d-phenylalaninamide (**6**)

A solution of silylated d-phenylalanine was prepared by addition of BSA (66 g, 325 mmol) during 1 h to a stirred suspension of d-phenylalanine (26.5 g, 161 mmol) in DCM (0.2 L) and stirring the mixture at room temperature for 17 h. The solution of *N*-pyrazine-carbonylimidazole was prepared by slow addition of *N*,*N*′-carbonyldiimidazole (34.0 g, 210 mmol) to a stirred suspension of pyrazinecarboxylic acid in DCM (0.4 L) at r.t. with subsequent reflux of the mixture for 17 h. To a reaction mixture, containing pyrazinecarboxylic acid imidazolide, a solution of silylated d-phenylalanine was added during 2 h, and the resulting solution was stirred for 17 h at r.t. After that the dichloromethane solution was washed with aqueous citric acid (80 g of citric acid monohydrate per 0.5 L of D.M. water), aqueous phase was extracted with ether (0.3 L), and the DCM and ether solutions were combined, washed with water (2×0.2 L) and dried over anhydrous sodium sulfate. The solvents were concentrated under vacuum to the volume of 0.1 L and cooled in the fridge. The amorphous precipitate formed was filtered off, washed with DCM and dried to obtain 27.2 g of yellow powder. Isol. yield 63 %. M.r. 169.5–170.9 °C. [Found: C, 61.85; H, 4.90; N, 15.32. C_14_H_13_N_3_O_3_ requires C, 61.99; H, 4.83; N, 15.49.]; [α]_D_^20^ −12.0 (*c* 1.0, MeOH). *m*/*z* (ESI-MS) 272 (M+H^+^). ^1^H NMR (360 MHz, acetone-d_6_, TMS): δ 9.22 (d, 1H, *J* = 1.4 Hz, CH_pyr_), 8.83 (d, 1H, *J* = 2.5 Hz, CH_pyr_), 8.63 (dd, 1H, *J* = 2.5, 1.44 Hz, CH_pyr_), 8.39 (br d, 1H, NH), 7.40–7.10 (m, 5H, CH_Ph_), 5.00 (m, 1H, αCH_Phe_), 3.34 (m, 2H, CH_2_Ph); ^13^ C NMR (90 MHz, DMSO-d_6_, TMS): δ 172.4, 162.6, 147.8, 144.1, 143.5, 143.4, 137.4, 129.1 (2C), 128.2 (2C), 126.5, 53.5, 36.3.

#### (1S,2S,3R,5S)-Pinane-2,3-dioxy *N*-pyrazinecarbonyl-d-phenylalanine-l-leucineboronate (*N*-{(1*R*)-3-Methyl-1-[(3a*S*,4*S*,6*S*,7a*R*)-3a,5,5-trimethylhexahydro-4,6-methano-1,3,2-benzodioxaborol-2-yl]butyl}-*Nα*-(pyrazin-2-ylcarbonyl)-d-phenylalaninamide, **8**)

(1*S*,2*S*,3*R*,5*S*)-Pinane-2,3-diol-(*R*)-1-ammonium-3-methylbutane-1-boronate trifluoracetate (15.0 g, 40 mmol), *N*-pyrazinecarbonyl-D-phenylalanine (10.7 g, 40 mmol), and TBTU (14.0 g, 44 mmol) were suspended in DCM (150 mL) under argon at −5 °C. A solution of DIPEA (20 mL, 123 mmol) in DCM (75 mL) was added dropwise over 2 h while stirring. The mixture was stirred at −5 °C for another 1 h and then at room temperature until the solution was clear. After that the solution was washed with water (75 mL), 10% aqueous K_2_CO_3_ (3×50 mL), 10% aqueous citric acid (75 mL), D.M. water (75 mL), and dried over anhydrous sodium sulfate. DCM was removed under vacuum, and the residue dissolved in ether (80 mL) and filtered through a pad of silica gel (180 mL of SiO_2_, eluent – ether). The solution obtained was evaporated under vacuum to yield 16.8 g of a glassy substance. Isol. yield 82%. M.r. 60.8–82.6 °C. M/*z* (ESI-MS): 367.28 (M − 152+H^+^), 519.39 (M+H^+^), 541.35 (M+Na^+^). ^1^H NMR (360 MHz, acetone-d_6_, TMS): δ 9.20 (d, 1H, *J* = 1.44 Hz, CH_pyr_), 8.82 (d, 1H, *J* = 2.2 Hz, CH_pyr_), 8.63 (m, 1H, CH_pyr_), 8.40 (br d, 1H, NH), 7.61 (br s, 1H, NH), 7.10–7.33 (m, 5H, CHPh), 4.93 (m, 1H, αCHPhe), 4.30 (dd, 1H, *J* = 8.6, 1.8 Hz, CH_pinane_–O), 3.2 (t, 2H, CH_2_Ph), 3.05–0.7 (m, 25H, αCH–B, 5CH3, 3CH_2_, 3CH); ^13^C NMR (75 MHz, CDCl_3_, TMS): δ 170.5, 162.5, 147.1, 144.0, 142.5, 136.3, 129.2 (2C), 128.2 (2C), 126.6, 85.3, 77.5, 53.7, 51.3, 39.8 (CH_2_), 39.4, 38.5 (CH_2_), 38.3, 37.9, 35.7, 35.4 (CH_2_), 28.4, 26.9, 26.1 (CH_2_), 25.9, 23.8, 22.7, 21.8.

#### N-Pyrazinecarbonyl-D-phenylalanine-L-leucineboronic acid (*N*,*N*′,*N*″-{Boroxin-2,4,6-triyltris[(1*R*)-3-methylbutane-1,1-diyl]}tri[*Nα*-(pyrazin-2-ylcarbonyl)-*L*-phenylalaninamide], **3**)

(1S,2S,3R,5S)-Pinanedioxy-*N*-pyrazinecarbonyl-d-phenylalanyl-l-leucineboronate (15.84 g, 31 mmol) and isobutylboronic acid (4.7 g, 46 mmol) were dissolved in methyl alcohol (120 mL). To this solution hexane (120 mL) and 1*N* hydrochloric acid (65 mL) were added. The resulting biphasic mixture was stirred for 17 h at r.t. The lower aqueous layer was separated, washed with hexane (3×15 mL), basified with sodium bicarbonate (*ca*. 6.7 g) to pH 5, and stirred for 30 min. The mixture was concentrated under vacuum and extracted with ethyl acetate (2×150 mL). The organic phase was washed with water (30 mL) and dried over anhydrous sodium sulfate. The solvent was removed under vacuum, and the residue crystallized from ethyl acetate (100 mL) at 60 °C. The precipitate was filtered off, washed with ethyl acetate and vacuum-dried to give 8.0 g of white powder. Isol. yield 68%. M.r. 186.2–195.7 °C (dec.). [α]_D_^20^ −6.0 (*c* 1.0, DMF). δH (600 MHz, DMSO-d_6_) 0.76 (s, 1H, CH_3_), 0.77 (s, 1H, CH_3_), 1.16, 1.33 (2m, 2H, CH_2_(CH_3_)_2_), 1.57 (m, 1H, CH(CH_3_)_2_), 2.63 (m, 1H, αCHB), 3.14 (m, 2H, CH_2_Ph), 4.94 (m, 1H, αCHPhe); 7.14–7.25 (m, 5H, Ph), 8.71 (m, 1H, NH), 8.85(d, 1H, CH_pyr_, *J* 2.4 Hz), 8.86–8.88 (m, 2H, CH_pyr_, NH), 9.09 (d, 1H, CH_pyr_, *J* 1.2 Hz); δC (150 MHz, DMSO-d_6_) 20.69, 22.57, 22.82, 25.00, 37.19, 40.07, 43.02, 51.69, 126.44, 128.05 (2C), 129.22 (2C), 136.87, 143.27, 143.46, 144.06, 147.66, 162.53, 170.25, 172.78.

#### Oxidation of bortezomib anhydride **2** (*N*-[(1*R*)-1-hydroxy-3-methylbutyl]-*Nα*-(pyrazin-2-ylcarbonyl)-l-phenylalaninamide, **4a**, (*N*-[(1*S*)-1-hydroxy-3-methylbutyl]-*Nα*-(pyrazin-2-ylcarbonyl)-l-phenylalaninamide, **4b**)

3% Aqueous hydrogen peroxide (10 mL) was added to a solution of bortezomib anhydride **2** (5.0 g, 13 mmol) in methanol (20 mL), and the resulting solution was stirred at r.t. for 3 h. The solvent was evaporated under vacuum, and the residue dissolved in diethyl ether (100 mL), filtered through the pad of silica gel (50 mL) and eluted with diethyl ether. The ethereal solution was evaporated to dryness to yield 3.9 g of the product as a mixture of epimeric alcohols **4a,b** in the ratio of 39:61. Isolated yield 83%. ^1^H and ^13^C spectra were in agreement with those reported for the separate diastereomers [8].

## Conclusions

We have studied the profile of impurities in bortezomib anhydride, synthesized by a previously developed convergent approach. Three organic impurities, including one which was previously unknown, were identified, synthesized and characterized.

## Figures and Tables

**Sch. 1:**
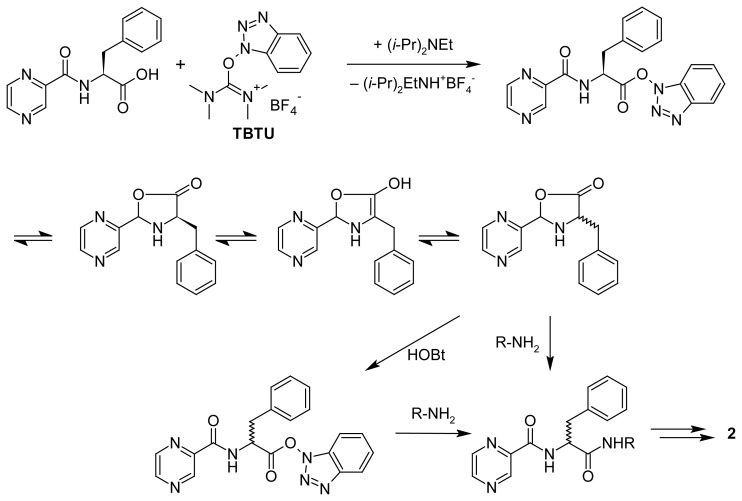


**Sch. 2:**